# N-Terminal Domain of Nuclear IL-1α Shows Structural Similarity to the C-Terminal Domain of Snf1 and Binds to the HAT/Core Module of the SAGA Complex

**DOI:** 10.1371/journal.pone.0041801

**Published:** 2012-08-06

**Authors:** Blanka Zamostna, Josef Novak, Vaclav Vopalensky, Tomas Masek, Ladislav Burysek, Martin Pospisek

**Affiliations:** 1 Department of Genetics and Microbiology, Faculty of Science, Charles University in Prague, Prague, Czech Republic; 2 Protean s.r.o., Dobra Voda u Ceskych Budejovic, Czech Republic; The John Curtin School of Medical Research, Australia

## Abstract

Interleukin-1α (IL-1α) is a proinflammatory cytokine and a key player in host immune responses in higher eukaryotes. IL-1α has pleiotropic effects on a wide range of cell types, and it has been extensively studied for its ability to contribute to various autoimmune and inflammation-linked disorders, including rheumatoid arthritis, Alzheimer’s disease, systemic sclerosis and cardiovascular disorders. Interestingly, a significant proportion of IL-1α is translocated to the cell nucleus, in which it interacts with histone acetyltransferase complexes. Despite the importance of IL-1α, little is known regarding its binding targets and functions in the nucleus. We took advantage of the histone acetyltransferase (HAT) complexes being evolutionarily conserved from yeast to humans and the yeast SAGA complex serving as an epitome of the eukaryotic HAT complexes. Using gene knock-out technique and co-immunoprecipitation of the IL-1α precursor with TAP-tagged subunits of the yeast HAT complexes, we mapped the IL-1α-binding site to the HAT/Core module of the SAGA complex. We also predicted the 3-D structure of the IL-1α N-terminal domain, and by employing structure similarity searches, we found a similar structure in the C-terminal regulatory region of the catalytic subunit of the AMP-activated/Snf1 protein kinases, which interact with HAT complexes both in mammals and yeast, respectively. This finding is further supported with the ability of the IL-1α precursor to partially rescue growth defects of *snf1*Δ yeast strains on media containing 3-Amino-1,2,4-triazole (3-AT), a competitive inhibitor of His3. Finally, the careful evaluation of our data together with other published data in the field allows us to hypothesize a new function for the ADA complex in SAGA complex assembly.

## Introduction

In the eukaryotic transcription process, the covalent acetylation of lysines on nucleosomal histone tails has been associated with DNA relaxation and enhanced transcriptional activity, whereas deacetylated chromatin regions have generally been proven to be transcriptionally inactive [1], [2]. The reversible process of histone acetylation is catalyzed by histone acetyltransferases (HATs) that, together with histone deacetylases (HDACs), play a pivotal role in the structural remodeling of the chromatin fiber. A wide range of proteins with intrinsic histone acetyltransferase activity has been identified, and most of these proteins are found within multisubunit HAT complexes whose specific HAT activity depends, to a large extent, upon the context of the associated subunits [3]. Histone acetyltransferases are generally well conserved throughout eukaryotes. Following the discovery of the first histone acetyltransferase Gcn5 in *Tetrahymena* [4], Gcn5-containing HAT complexes have been identified in other organisms, including yeast [5] and humans [6].

The 1.8-MDa *Saccharomyces cerevisiae* SAGA (Spt-Ada-Gcn5 acetyltransferase) complex [7] is the most studied HAT complex and one of the main transcriptional co-activators in yeast. Originally, the SAGA complex was described in yeast, but it has subsequently been shown to be evolutionarily conserved from yeast to humans and has rapidly become the epitome of eukaryotic HAT complexes [8], [9]. Recently Lee and co-workers suggested that the SAGA complex consists of and assembles from five distinct modules: HAT/Core (histone acetyltransferase catalytic core), DUB (deubiquitinylation module), an ADA module, SA_SPT (SAGA-associated suppressors of Ty) and SA_TAF (SAGA-associated TATA-binding protein-associated factors) [10]. Gcn5 is the catalytic subunit that mediates the acetyltransferase function, and its activity is modulated by the associated proteins Ada2 and Ada3 [7], [11], [12] forming with Sgf29 altogether the HAT/Core module [10]. Another SAGA subcomplex, the SA_SPT module, is composed of Ada1 [13], Ada5/Spt20 [14], Spt7 (required for complex integrity), the Spt3 and Spt8 proteins (both involved in the interaction with the TATA-binding protein) [15], [16], [17], [18] and Tra1, which is a homologue of the mammalian transcriptional co-activator TRRAP [10], [19], [20]. A subset of essential Taf proteins represented by Taf5, Taf6, Taf9, Taf10 and Taf12 [21] forms the SAT_TAF module [10]. Furthermore, Ubp8, Sus1, Sgf11 and Sgf73 have been shown to be components of the DUB module [22], [23], [24]. The Chd1 protein has also been found to be associated with the SAGA complex [25], but this result was not confirmed by another study [10].

Another yeast histone acetyltransferase complex is SAGA-related, yet less studied, the 0.8-MDa ADA complex. The catalytic subunit of ADA is Gcn5 which together with Ada2, Ada3 [7] and Sgf29 [10] forms the HAT/Core module that is identical in the SAGA and ADA complexes. A molecular marker of the ADA complex is Ahc1, which has been suggested to be essential for ADA complex integrity [26]. Recently, another protein that is typical of the ADA complex was identified and termed Ahc2 [10]. Presumably, ADA contains none of the Spt proteins, and Ada1 and Tra1 are not part of this complex either [19], [26], [27]. Although certain subunits are shared by the SAGA and ADA HAT complexes, these complexes do not appear to play the same role in yeast cells. In contrast to the SAGA complex, ADA does not interact with the acidic activators Gcn4 and VP16 [28], and due to the absence of the Spt proteins, ADA most likely does not bind to the TATA-binding protein (TBP). Both HAT complexes also show distinct acetylation pattern, which appears to be broader in case of the SAGA complex [29]. The ADA complex has been suggested to be either a SAGA subcomplex [30] or, more recently, rather a distinct HAT complex with specific functions and activities [26].

Interleukin-1α (IL-1α) is a proinflammatory cytokine and a key player in host immune responses in higher eukaryotes. IL-1α represents a molecule with pleiotropic effects on a wide range of cell types [31] and has been extensively studied for its ability to contribute to various human autoimmune and inflammation-linked disorders, including rheumatoid arthritis, Alzheimer’s disease, systemic sclerosis and cardiovascular disorders [32], [33], [34], [35]. The 31-kDa IL-1α precursor (pre-IL-1α) is proteolytically cleaved by calpain to release the 17-kDa mature IL-1α (IL-1αMat) and the 16-kDa N-terminal portion of IL-1α (IL-1αNTP). Due to the nuclear localization sequence (NLS) within the N-terminal portion of the molecule (amino acids 79–86) [36] pre-IL-1α and IL-1αNTP are commonly found in the nucleus. Multiple studies have reported an IL-1α interaction with nuclear proteins, including the HAX-1 protein [37], the growth suppressor protein necdin [38] and the components of the RNA splicing apparatus [39]. We previously studied IL-1α in the *Saccharomyces cerevisiae* model organism and discovered the genetic interaction between nuclear IL-1α and the yeast SAGA HAT complex. We further confirmed these results in mammalian cells by demonstrating that pre-IL-1α physically and functionally associates with the p300/PCAF/Gcn5 HAT complexes via its N-terminal peptide [40]. These results proved that yeast is an excellent model for the study of IL-1α nuclear signaling that is mediated by its interaction with the histone acetyltransferase complexes. Despite the increased attention of the scientific community and numerous studies on the nuclear function of pre-IL-1α, its direct nuclear target and functions in the control of gene expression remain to be uncovered.

In this study, we took advantage of the yeast model organism and bioinformatics approaches to extend our understanding of the nuclear interaction between pre-IL-1α and histone acetyltransferase complexes. We demonstrate that pre-IL-1α physically interacts with the HAT/Core module of SAGA complex and suggest possible competition of pre-IL-1α and AMP-activated protein kinase for the same binding site. Our results also suggested a new model of SAGA complex assembly. In contrast to previous assumptions, we show that the ADA complex may represent an intermediate stage in SAGA complex assembly and that the Ahc1 protein may play a key role in this process.

## Materials and Methods

### *Saccharomyces cerevisiae* Strains and Plasmids

All of the strains used in this study are listed in Table 1. The standard W303-1a strain was kindly provided by Beate Schwer. The strains *snf1-108* and *snf1*Δ were kindly provided by Min-Hao Kuo. Yeast strains harboring the TAP-tagged proteins Gcn5, Spt7, Spt8, Ada1, Ada2, Ada3 and Ahc1 were derived from BY4741 [41], and they were kindly provided by Zuzana Storchova. The *SPT7* (YBR081C), *GCN5* (YGR252W), *AHC1* (YOR023C) and *AHC2* (YCR082W) genes were deleted from the chromosomes of the respective yeast strains using *loxP-kanMX-loxP* and/or *loxP-Leu2-loxP* cassette [42]. The nucleotide sequences of the primer sets used for the amplification of the gene disruption cassettes are summarized in Table 2. Successful gene disruptions were verified by PCR and western blotting.

**Table 1:** Yeast strains used in this study.

*S. cerevisiae* strain	Genotype	Reference/Source
W303-1a	MATa; *ade2-1; can1-100; his3-11,15; leu 2-3,112; trp1-1; ura3-1*	ATCC 208352
TAP/Gcn5	MATa; *leu2-0; met15-0; ura3-0; GCN5-TAP::HIS3*	[41]
TAP/Spt7	MATa; *leu2-0; met15-0; ura3-0; SPT7-TAP::HIS3*	[41]
TAP/Spt8	MATa; *leu2-0; met15-0; ura3-0; SPT8-TAP::HIS3*	[41]
TAP/Ada1	MATa; *leu2-0; met15-0; ura3-0; ADA1-TAP::HIS3*	[41]
TAP/Ada2	MATa; *leu2-0; met15-0; ura3-0; ADA2-TAP::HIS3*	[41]
TAP/Ada3	MATa; *leu2-0; met15-0; ura3-0; ADA3-TAP::HIS3*	[41]
TAP/Ahc1	MATa; *leu2-0; met15-0; ura3-0; AHC1-TAP::HIS3*	[41]
TAP/Ahc1,*gcn5*Δ	MATa; *leu2-0; met15-0; ura3-0; AHC1-TAP::HIS3; gcn5Δ::kanMX*	this study
TAP/Spt8,*gcn5*Δ	MATa; *leu2-0; met15-0; ura3-0; SPT8-TAP::HIS3; gcn5Δ::kanMX*	this study
TAP/Gcn5,ahc1Δ	MATa; *leu2-0; met15-0; ura3-0; GCN5-TAP::HIS3; ahc1Δ::kanMX*	this study
TAP/Spt8,*ahc1*Δ	MATa; *leu2-0; met15-0; ura3-0; SPT8-TAP::HIS3; ahc1Δ::kanMX*	this study
TAP/Spt7,*ahc1*Δ	MATa; *leu2-0; met15-0; ura3-0; SPT7-TAP::HIS3; ahc1Δ::kanMX*	this study
TAP/Spt8,*spt7*Δ	MATa; *leu2-0; met15-0; ura3-0; SPT8-TAP::HIS3; spt7Δ::kanMX*	this study
TAP/Ahc1,*spt7*Δ	MATa; *leu2-0; met15-0; ura3-0; AHC1-TAP::HIS3; spt7Δ::LEU2*	this study
TAP/Gcn5,*ahc2*Δ	MATa; *met15-0; ura3-0; GCN5-TAP::HIS3; ahc2Δ::LEU2*	this study
TAP/Spt8,*ahc2*Δ	MATa; *met15-0; ura3-0; GCN5-TAP::HIS3; ahc2Δ::LEU2*	this study
TAP/Spt7,*ahc2*Δ	MATa; *met15-0; ura3-0; GCN5-TAP::HIS3; ahc2Δ::LEU2*	this study
*snf1-108*	MATa; *trp1; leu2-3,112; ura3-52; snf1-108::TRP1*	[48]
*snf1*Δ	MATa; *trp1, leu2-3,112; ura3-52; snf1Δ::LEU2*	[48]

**Table 2:** Nucleotide sequences of the primers used for the *loxP-kanMX-loxP* and *loxP-Leu2-loxP* gene disruption cassette amplification.

Name	Sequence
Gcn5Fwd	AGAAGGCACGTAAATAATCTTAAACACTTATGGGCAGCAAAAAATGCGTCTTTCTTCCCCGTACGCTGCAGGTCGACAACC
Gcn5Rev	GAAGCTGCAGAAAGTCCAGAAGAAGCGGATGTTGAAATGTCATAAATATAGTTACACGACTCACTATAGGGAGACCG
Spt7Fwd	GCTCTTATCTTTGAGAGAAACAACGGTAGTACTTAAAGGCTACGAATAAGCCAATTTCCGTACGCTGCAGGTCGACAACC
Spt7Rev	CAGATCCAAGTAATGAAAAGTGAATGTACACTCGTAAGTCTTCAATTATGTTAGATGTACGACTCACTATAGGGAGACCG
Ahc1Fwd	ATACAATCCACTTTTTCTTCCAAAGCGACAAATTAGGTCAGAAAACCAACCGTACGCTGCAGGTCGACAACC
Ahc1Rev	ATAGCCGTAGGAAGAGGGAAAAACGAACAGGGAAGAAGCAGGAAGAGCAGACGACTCACTATAGGGAGACCG
Ahc2-delALL-F	CGCTTACAAGATCAGTACTAGCGTGTACCAGTCTATTTAAACTGGACGCATAGGCCACTAGTGGATCTG
Ahc2-delALL-R	ATATATATAAATATATGTTTAAAAATGTCCATAGCCGCTATTTGACAGCTGAAGCTTCGTACGC

To study pre-IL-1α in yeast cells, genes encoding either human full-length (amino acids 1-271; pre-IL-1α) or mature (amino acids 113-271; IL-1αMat) IL-1α were inserted as an N-terminal fusion with a Flag tag into the yeast expression plasmid pYX133 and/or pYX212 (Ingenius). To determine the subcellular localization of IL-1α in yeast cells, we inserted pre-IL-1α and IL-1αMat into the pUG36 plasmid (GenBank: AF298791.1, a gift from J. H. Hegemann) to enable the expression of both IL-1α variants fused with the yeast-enhanced green fluorescent protein (yGFP). All of the yeast transformations were performed with the one-step LiCl method [43]. The cells were grown in a shaker at 28°C in drop-out synthetic minimal medium (SD) without tryptophan (plasmids derived from pYX133) or uracil (plasmids derived from pYX212 and pUG36) to ensure plasmid maintenance.

### Fluorescence Microscopy of Yeast Cells

Cells harboring the empty pUG36 or pUG36 plasmids containing either pre-IL-1α or IL-1αMat were grown in SD medium lacking methionine and uracil until they reached OD_600_ = 0.8. The cells were sedimented with centrifugation at 3,000× g for 5 min and resuspended in 1 ml of medium. A total of 100 µl of formaldehyde was added to the tubes, and the samples were incubated for 1 h at room temperature. The fixed cells were washed in 0.5 ml of KPO_4_/sorbitol solution (60 ml 2 M sorbitol, 10 ml 1 M potassium phosphate and 30 ml distilled water) and resuspended in 300 µl of KPO_4_/sorbitol. A total of 2 µl of the cell suspension was mixed with 2 µl of mounting medium containing DAPI (100 mg p-phenylenediamine in 10 ml of PBS, 90 ml glycerol and 2.25 µl of fresh DAPI solution at 1 mg/ml in distilled water), and the samples were observed under a fluorescence microscope.

### Co-immunoprecipitation from Yeast Cells

For the co-immunoprecipitation experiments, we used 100 to 200 ml of yeast culture that was grown to OD_600_ = 0.8-1.2. The cells were harvested with centrifugation, washed with ice-cold distilled water and lysed using nondenaturing lysis buffer (1% Triton X-100, 50 mM Tris-HCl, pH 7.4, 300 mM NaCl, 5 mM EDTA and 0.02% sodium azide) supplemented with protease inhibitors (Complete Mini, Roche) and 0.45 mm glass beads. The lysates were clarified with centrifugation at 20,000× *g* for 20 min at 4°C, and the resulting supernatants were pre-cleared with 20 µl of protein G agarose (Exalpha Biologicals) for 1 h at 4°C. Co-immunoprecipitations were performed using 1 µl of mouse monoclonal anti-Flag antibody (clone M2; Sigma Aldrich) and 30 µl of protein G agarose with gentle mixing overnight at 4°C. The immunoprecipitated complexes were dissolved in 2X sample loading buffer (0.1 M Tris-HCl, pH 6.8, 20% glycerol, 2% β-mercaptoethanol, 4% SDS and 0.04% bromophenol blue), incubated at 95°C for 5 mins and subjected to SDS-PAGE.

### Western Blotting

SDS-PAGE gels were electroblotted onto a nitrocellulose membrane (NC2; Serva). The membranes were blocked in 5% milk (Sunar Complex 1; Hero) in a TBS-Tween solution (50 mM Tris, 150 mM NaCl and 0.5% Tween-20) and incubated with a rabbit anti-calmodulin-binding peptide antibody (anti-CBP, 1∶2,500; ICL) overnight at 4°C. After washing in TBS-Tween and blocking with 5% milk, the membranes were incubated with a swine anti-rabbit HRP-conjugated antibody (1∶5,000; Sevapharm). Finally, the membranes were immersed in a luminol detection solution and exposed to X-ray film (Medix; FOMA). To confirm the expression and the successful immunoprecipitation of pre-IL-1α or IL-1αMat, a mouse monoclonal anti-Flag antibody (1∶5,000; Sigma Aldrich) and goat anti-mouse HRP-conjugated antibodies (1∶5,000; Santa Cruz Biotechnology) were used.

### Bioinformatic Analyses

The Robetta server at http://robetta.bakerlab.org was used to predict the IL-1αNTP 3-D structure. The Dali server at http://ekhidna.biocenter.helsinki.fi/dali_server was used for structure similarity searches. The proteins used throughout the analyses were as follows: human pre-IL-1α (NCBI Reference Sequence: NP_000566.3), SNF-1 (PDB ID: 3T4N) and AMPK (PDB ID: 2V92).

## Results

### Evaluating Yeast as a Model for the Study of IL-1α Nuclear Signaling

In our previous study, we demonstrated that IL-1αNTP fused to a Gal4p DNA-binding domain (Gal4BD) possessed transactivation potential, and due to interference with host transcription, it exhibited a toxic phenotype in *S. cerevisiae.* The Gal4BD/IL-1αNTP fusion toxicity was completely abrogated in strains harboring *GCN5, ADA2, ADA3* and *SPT7* gene deletions but not in a strain containing an *AHC1* gene deletion, thus resembling the Ada^-^ phenotype. Importantly, the findings on the genetic and functional interaction of the Gal4BD/IL-1αNTP fusion with the yeast SAGA complex were recapitulated in a mammalian model, in which we demonstrated that both human pre-IL-1α and IL-1αNTP but not the mature IL-1α functionally and physically interacted with the human and mouse Gcn5, p300 and PCAF histone acetyltransferase complexes [40]. Similarly, Werman and co-workers also described the activation of transcription at GAL4-UAS promoter by Gal4BD/pre-IL-1α chimera in mammalian cells and suggested pre-IL-1α as a proinflammatory transcription activator [44].

In this study, we extend our previous work in the yeast model to dissect the interactions between pre-IL-1α and the SAGA complex in more detail through immunoprecipitation with pre-IL-1α and mature IL-1α as a major experimental approach. Initially, we determined whether pre-IL-1α localizes to the yeast nucleus in a similar manner as in mammalian cells. The human IL-1α precursor contains a nuclear localization sequence within the N-terminal portion of the molecule [36]. During the process of IL-1α maturation, the N-terminal portion is removed by calpain, and mature IL-1α lacks the NLS. Consequently, in mammalian cells, the IL-1α precursor is localized to the cell nucleus, whereas mature IL-1α resides in the cytoplasm.

**Figure 1:**
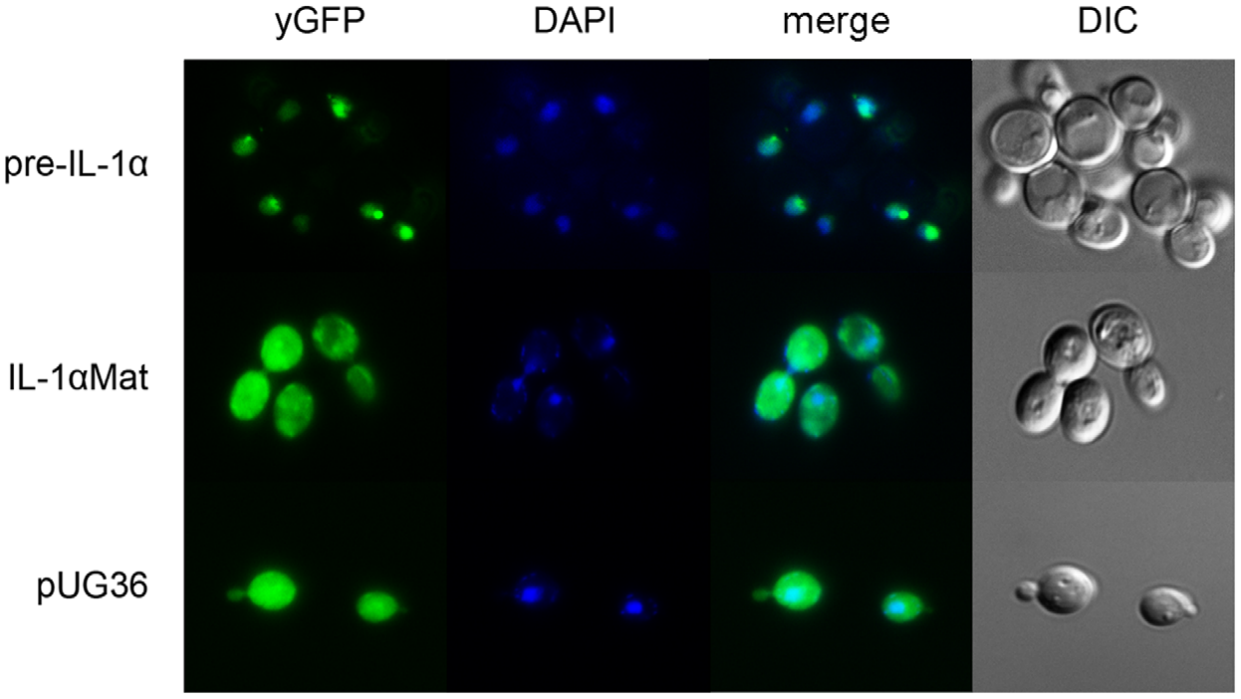
Subcellular localization of pre-IL-1α and IL-1αMat in *Saccharomyces cerevisiae.* The IL-1α precursor (pre-IL-1α) is exclusively localized in the nucleus of yeast cells, which is in contrast to the observed cytoplasmic localization of mature IL-1α (IL-1αMat). Control cells (ctrl) carry the empty pUG36 vector. The cell nuclei are stained with DAPI.

To determine whether the subcellular localization of human pre-IL-1α and IL-1αMat heterologously expressed in *S. cerevisiae* corresponds to the situation in mammalian cells, we produced IL-1α proteins tagged with yGFP (yeast-enhanced GFP) in yeast and performed analysis with fluorescence microscopy. The cDNA corresponding to IL-1αMat and pre-IL-1α were inserted into the yeast expression vector pUG36 (J. H. Hegemann, GenBank: AF298791.1), and the constructs were introduced into the W303-1A *S. cerevisiae* strain. The results confirmed the nuclear localization of pre-IL-1α in yeast cells because the yGFP fluorescence co-localized with DAPI-stained nuclei (Figure 1). In contrast, the localization of both yGFP in control cells bearing the empty pUG36 vector and IL-1αMat was cytoplasmic. Therefore, the subcellular localization of both IL-1α proteins in *S. cerevisiae* is analogous to the localization pattern in mammalian cells, and the nuclear trafficking of the IL-1α precursor appears to be conserved in yeast cells.

**Figure 2:**
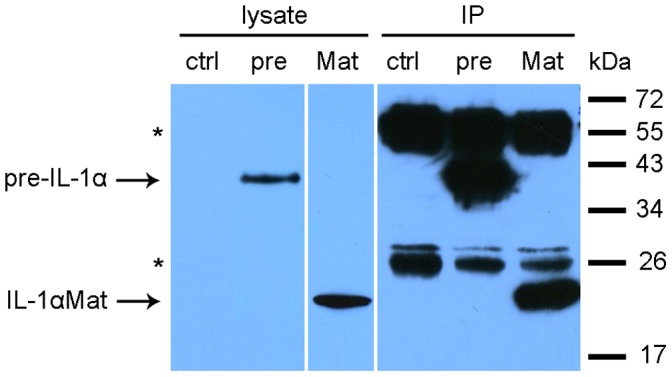
Heterologous expression and immunoprecipitation of the IL-1α proteins in yeast. “pre” represents the IL-1α precursor produced from the plasmid 212-pre-Flag, and “Mat” represents mature interleukin-1α produced from 212-Mat-Flag. A yeast strain transformed with empty vector was used as the control (ctrl). Immunoprecipitation (IP) and western blotting was performed using an anti-Flag antibody recognizing the Flag tag at the N-terminus of the IL-1α proteins. Asterisks indicate the bands corresponding to the heavy and light chains of the anti-Flag antibody. Molecular size marker positions are shown at the right.

In order to find suitable conditions for co-immunoprecipitation of yeast HAT complexes via interleukin-1α as their interacting partner, we prepared a set of yeast low- and high-copy number expression vectors allowing us to produce intracellularly either pre-IL-1α or the matured interleukin-1α with various N- and C-terminal epitopes. For the rest of this study we chose N-terminal tagging of the interleukin-1α peptides by Flag epitope and vectors based on yeast expression multicopy plasmid pYX212 (Ingenius), which contains strong constitutive triose phosphate isomerase promoter. The expression of the Flag-tagged IL-1α precursor and Flag-tagged mature IL-1α produced from the plasmids 212-pre-Flag and 212-Mat-Flag in yeast cells and a possibility to immunoprecipitate both proteins from a yeast cell lysate with low background were verified by western blotting (Figure 2).

During our study of yeast strains producing the IL-1α peptides, we observed that some deletion yeast strains producing pre-IL-1α show decreased viability after prolonged storage on agar plates and that expression plasmids coding for the IL-1α precursor were more rapidly eliminated from the cells than control plasmids. We hypothesize that intracellular production of pre-IL-1α interferes with some important intracellular pathways or structures and thus influences yeast cell fitness. However, we did not observe any remarkable differences in growth in liquid selective media between yeast strains carrying empty plasmids or derived corresponding plasmids encoding either pre-IL-1α or mature IL-1α (data not shown).

### Bioinformatic Analysis Suggests that IL-1αNTP can Bind to the AMP-activated Protein Kinase-binding Site of the Histone Acetyltransferase Complex

**Figure 3:**
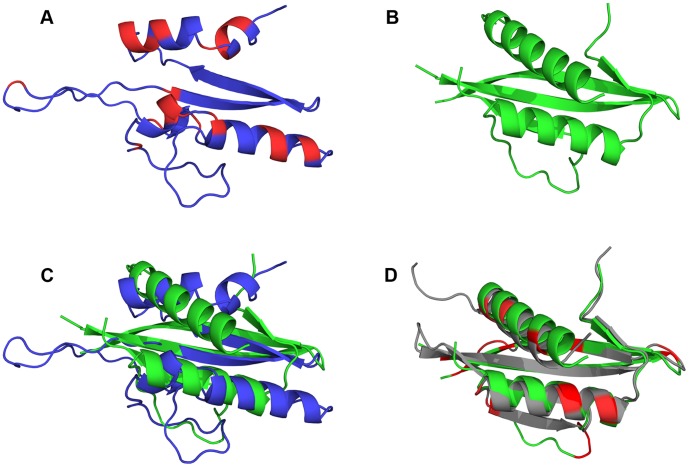
The structure of IL-1αNTP resembles the C-terminal portion of the catalytical subunit of the eukaryotic AMP-activated protein kinase. (A) Prediction of the 3-D structure of the first N-terminal 112 amino acid residues of the IL-1α precursor (IL-1αNTP). Acidic amino acid residues are depicted in red. (B) The 3-D structure of the INL domain of the yeast Snf1 protein kinase (PDB ID: 3T4N). (C) A superimposition of IL-1αNTP (blue) and the C-terminal INL domain of the yeast Snf1 protein kinase (green). (D) A superimposition of the INL domains of yeast Snf1 (green, PDB ID: 3T4N) and rat AMP-activated protein kinase (grey, PDB ID: 2V92). Acidic amino acid residues are depicted in red tones. See also File S1 for cordinates of IL-1αNTP prediction and File S2 for primary sequences of all proteins used in this analysis.

Previously, we provided evidence that both N-terminal and C-terminal helices of IL-1αNTP are important for its nuclear function and interaction with both the mammalian and yeast histone acetyltransferase complexes [40]. The structure of the SAGA complex is evolutionarily conserved from yeast to mammals. If one expects a similar or identical mode of interaction between human IL-1αNTP and both the yeast SAGA complex and its human analog, one would also expect that yeast cells may naturally contain some peptide structures similar to IL-1αNTP. To test this hypothesis, we performed structure similarity searches with IL-1αNTP against known structures in the Protein Data Bank (PDB). The structures of IL-1αNTP and full-length pre-IL-1α have yet not been determined. Therefore, we employed the Robetta server [45] to obtain *ab initio* models of IL-1αNTP. For prediction, we used the first 112 N-terminal amino acid residues of human pre-IL-1α (NCBI Reference Sequence: NP_000566.3). We obtained five predicted structures, which we used for structure similarity searches using the Dali server at the Institute of Biotechnology, University of Helsinki [46]. The best-scored and only meaningful hit returned by one of the IL-1αNTP structure predictions was the C-terminal regulatory domain of the Snf1 kinase alpha subunit (Figure 3). The Snf1 kinase is a founding member of the AMP-activated protein kinase family (AMPK). Yeast with a deleted *SNF1* gene are viable; however, its loss leads to reduced fitness under various stress conditions, an inability to grow on sucrose, galactose, maltose, melibiose and non-fermentable carbon sources and sporulation defects (*Saccharomyces* Genome Database). Snf1 has been shown to functionally and physically interact with members of the SAGA complex (Gcn5, Sgf73, Spt3, Spt8 and Ubp8) and Ahc1, an identifying member of the ADA complex [47], [48], [49]. Snf1 phosphorylates Gcn5 and can undergo deubiquitination by Ubp8, which is another member of the SAGA complex. As depicted in Figure 3C, an overlay of IL-1αNTP and the Snf1 C-terminal regulatory domain demonstrates the high extent of their structural similarity in 3-D. The AMPK/Snf1 protein family displays a high degree of similarity in amino acid sequences across all eukaryotes [50], [51]. We investigated whether the AMPK/Snf1 C-terminal Interleukin-1αNTP-Like domain (INL domain) was structurally similar in yeast and mammals. We superimposed the INL domains of yeast Snf1 (PDB ID: 3T4N) and the rat AMP-activated protein kinase (PDB ID: 2V92), and this overlay revealed that both structures were almost identical, including the positions of some acidic residues. The AMPK/Snf1 INL domains span amino acid residues 406 to 559 and 505 to 630, respectively. See also File S1 for cordinates of IL-1αNTP prediction and File S2 for primary sequences of all proteins used in this analysis.

**Figure 4:**
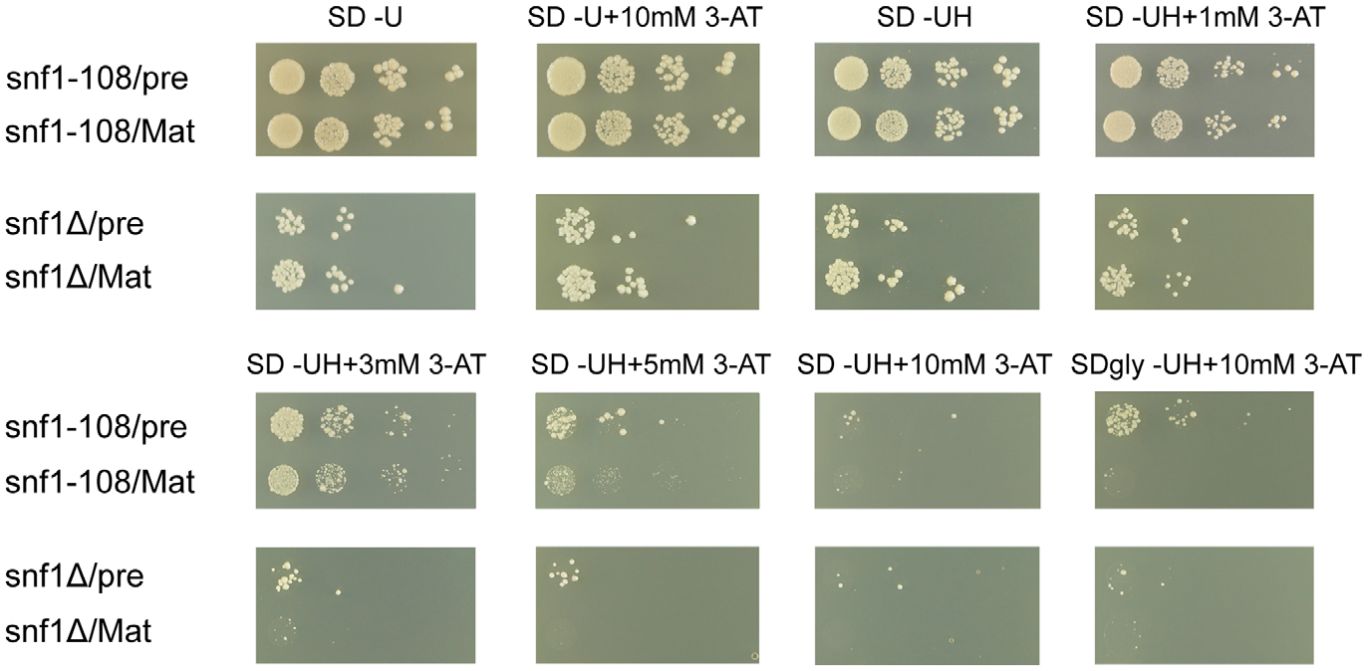
The interleukin-1α precursor suppresses hypersensitivity of *snf1*Δ strain to 3-Amino-1,2,4-triazole (3-AT), a competitive inhibitor of the His3 imidazoleglycerol-phosphate dehydratase. This suppressive property of pre-IL-1alpha (pre) is more profound on SD media containing glycerol/ethanol as a carbon source (SDgly) in case of the *snf1-108* strain carrying incomplete *SNF1* deletion. Mature interleukin-1alpha (Mat) is not able to rescue the 3-AT hypersensitivity of both *snf1*Δ and *snf1-108* strains and was used as a control. We did not observe any differences in growth between strains producing pre-IL-1alpha and mature IL-1alpha on SD agar plates which did not contain and/or contain only a minute amount of 3-AT. This is an example of 3 independent biological experiments.

To investigate if observed structural similarity between IL-1αNTP and the Snf1 C-terminal regulatory domain may have some biological relevance, we decided to test whether the pre-IL-1α production can cause some detectable phenotype in *snf1* mutant strains. We employed two deletion mutant strains, kindly obtained from Min-Hao Kuo, containing alleles *snf1*Δ and *snf1-108,* which was truncated after the 108th codon. Growth defects on agar plates containing 3-Amino-1,2,4-triazole (3-AT), which is a competitive inhibitor of His3, were reported about both mutant strains [48]. We observed impaired growth on 3-AT plates of both *snf1*Δ and *snf1-108* strains, similarly as described by Liu and co-authors, however, the production of pre-IL-1α slightly suppressed this growth defect if compared to strains producing mature IL-1α or strains containing an empty control plasmid only. Because Snf1 is a key regulator of yeast growth in non-fermentable media, we wanted to perform the same tests on glycerol agar plates. Surprisingly, pre-IL-1α significantly rescued growth defects of the *snf1-108* strain but not the *snf1*Δ mutant strain on glycerol agar plates containing 3-AT. These results provide another evidence supporting our structural model, and because of the known role of Snf1 and SAGA complex in the regulation of transcription initiation at *HIS3* promoter [48], [52], point to the possibility of a competition between pre-IL-1α and Snf1/AMPK for the same binding sites in HAT complexes (Figure 4).

### The IL-1α Precursor Physically Interacts with the HAT/Core Module Shared by the SAGA and ADA Complexes

**Figure 5:**
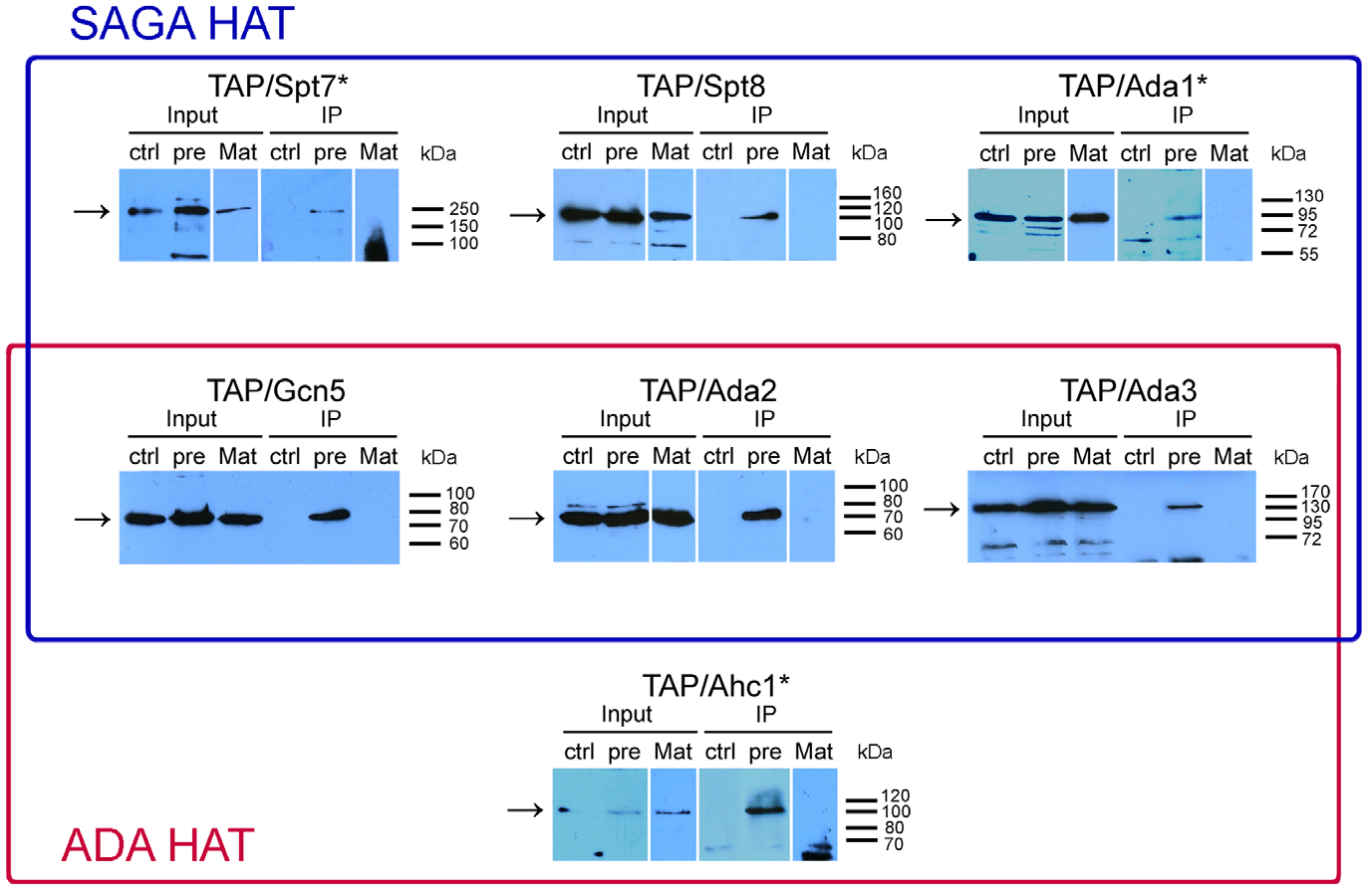
The SAGA and ADA complex subunits co-purify as a part of the IL-1α precursor-binding complex. Co-immunoprecipitation experiments with an anti-Flag antibody using yeast strains from a TAP tag library transformed with IL-1α expression vectors revealed that both of the HAT complexes bound pre-IL-1α (pre) but not mature IL-1α (Mat). Control cells (ctrl) carry the empty plasmid pYX212. Western blotting was performed using an anti-CBP antibody that recognizes the TAP tag at the C-terminus of the respective HAT complex subunits. For each line of the IP experiment, 1.4 mL of the cell lysate prepared from 5.10^8^ yeast cells in average was used. Inputs contain 16.7 µL of the corresponding lysates taken before the lysates were used for immunoprecipitation.

Our previous results showed that the nuclear localization and interaction of the IL-1α precursor with histone acetyltransferase complexes are similar in yeast and human cells (above and [40]). However, our structure similarity searches and serial drop tests with *snf1*Δ strains suggested that the IL-1α precursor may interact not only with the SAGA complex but also with the ADA complex because the Snf1 kinase was genetically shown to interact with Ahc1, the structural subunit of the ADA complex [47]. This interaction would be surprising because the expression of the Gal4BD/IL-1αNTP fusion in yeast induces a classical Ada^-^ phenotype, and the *ahc1* null mutation, in contrast to *gcn5*Δ, *ada2*Δ, *ada3*Δ *and spt7*Δ, did not rescue the toxicity of Gal4BD/IL-1αNTP overproduction [40]. To examine this interaction, we took advantage of the power of yeast model and employed a yeast TAP fusion library [41]. Plasmids 212-pre-Flag and 212-Mat-Flag were separately introduced into *S. cerevisiae* BY4741 strains expressing selected TAP-tagged subunits of either the SAGA or ADA complex [41] and subsequently a set of co-immunoprecipitation experiments using an anti-Flag antibody against the Flag-tagged IL-1α precursor and the Flag-tagged mature IL-1α as a control was performed (Figure 5).

Interestingly, in agreement with our IL-1α structure modeling and *snf1*Δ phenotype testing results, these co-immunoprecipitation experiments revealed that all of the SAGA and ADA complex subunits tested, including their structural subunits Spt7 and Ahc1, respectively, were present in protein complexes that bound pre-IL-1α. In contrast, none of these subunits bound mature IL-1α, although western blotting confirmed the expression of the proteins in IP lysates and the successful immunoprecipitation of mature IL-1α (Figure S1). These results are further supported by the fluorescence microscopy experiments depicted in Figure 1 that show the differential subcellular localization of mature IL-1α and the IL-1α precursor in yeast.

Our results strongly suggest that the IL-1α precursor binds to the HAT/Core module consisting of Ada2, Ada3, Gcn5 and Sgf29 because these subunits are the only polypeptides shared by the SAGA and ADA complexes [10].

### Gene Knock-out Analysis of pre-IL-1α Binding to the HAT Complexes Refined its Interaction with the HAT/Core Module and Suggested a Mutually Exclusive Role for Spt7 and Ahc1 in SAGA Complex Assembly

To refine our results and further elucidate the interactions between IL-1α and the yeast histone acetyltransferase complexes, we performed a series of gene disruptions in a set of strains that produce TAP fusions of the SAGA and ADA histone acetyltransferase complex subunits. The genes *GCN5* (catalytic subunit of both the SAGA and ADA complexes), *SPT7* (maintains the integrity of SAGA), *AHC1* (maintains the integrity of ADA) and *AHC2* (a recently identified specific subunit of the ADA complex) were replaced with the *kanMX* and/or *Leu2* gene disruption cassettes [42]. Successful gene deletions were confirmed with PCR.

**Figure 6:**
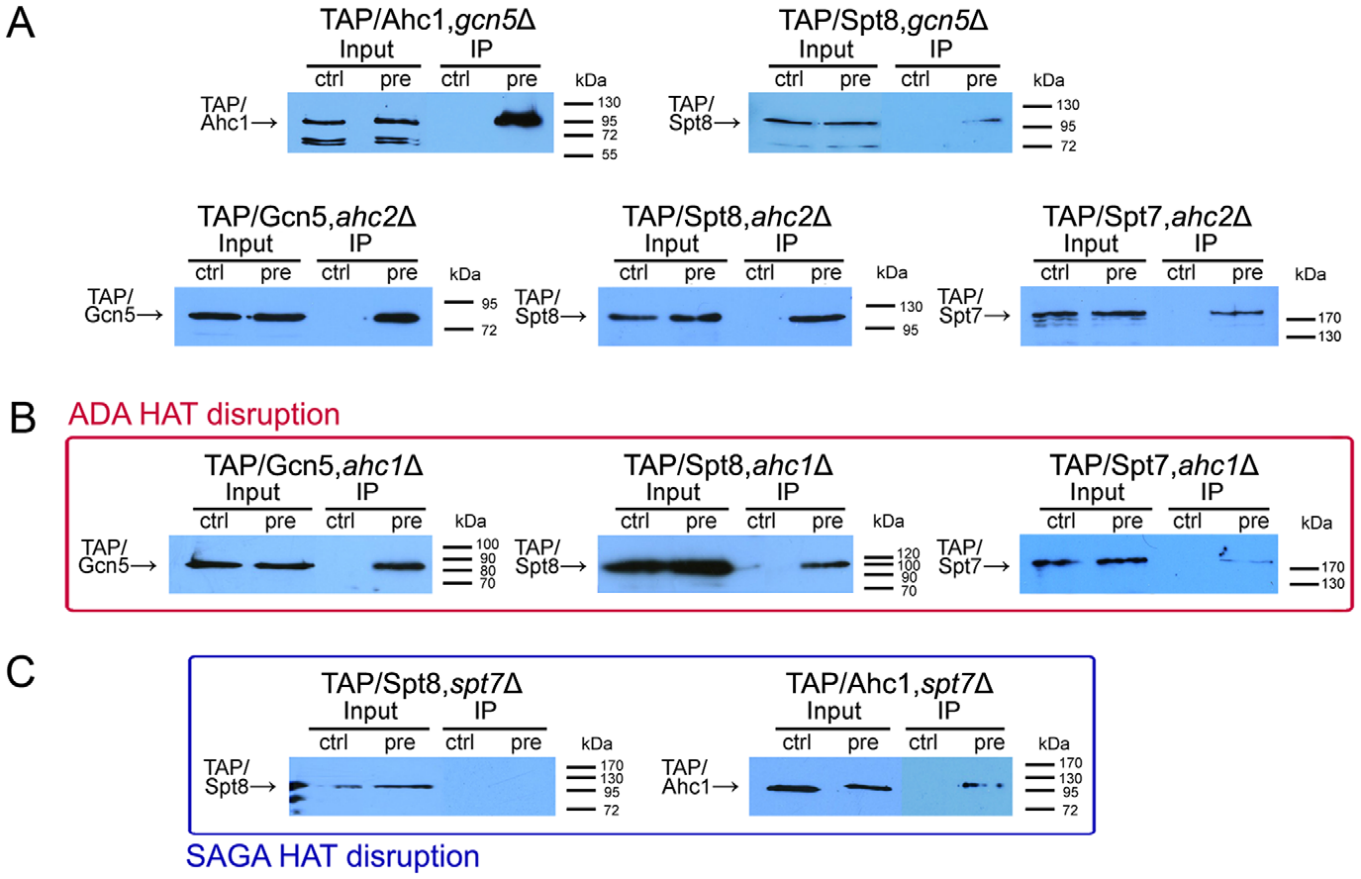
Disruption of the SAGA and ADA complexes confirmed binding of the IL-1α precursor to the HAT/Core module and suggested the mutually exclusive role of Spt7 and Ahc1 in SAGA complex assembly. Co-immunoprecipitation was performed using yeast lysates with an anti-Flag antibody that recognizes the Flag tag at the N-terminus of the IL-1α precursor. Western blotting was performed with an anti-CBP antibody which identifies the TAP tag at the C-terminus of the respective HAT complex subunits. (A) Gcn5 does not bind to pre-IL-1α, and because it is not required for SAGA or ADA complex integrity, its deletion has no effect on Ahc1 or Spt8 co-immunoprecipitation with the IL-1α precursor (pre). Deletion of the AHC2 gene doesn’t impair co-IP of pre-IL-1α with Gcn5, Spt8 and Spt7. (B) The disruption of the ADA HAT complex did not affect the co-immunoprecipitation of Gcn5 and Spt8 with IL-1α. However, the interaction between Spt7 and the IL-1α precursor was significantly weakened. In experiments with TAP/Spt7,*ahc1*Δ strain, we received either no or very low signal (the latter is depicted) of TAP-tagged Spt7, with a success rate 3∶1, respectively. (C) The disruption of the SAGA complex abolished the interaction between Spt8 and the IL-1α precursor but had no effect on Ahc1 binding to the IL-1α precursor. Control cells (ctrl) carry the empty plasmid pYX212. For each line of the IP experiment, 3.5 mL of cell lysate prepared from 20.10^8^ yeast cells in average was used, except TAP/Spt8,*gcn5*Δ, TAP/Spt8,*ahc1*Δ, TAP/Spt8,*spt7*Δ and TAP/Gcn5,*ahc1*Δ strains, where 1.3 mL of cell lysates from 9.10^8^ yeast cells each were applied. Inputs contain 16.7 µL of the corresponding lysates taken before the lysates were used for immunoprecipitation.

The *GCN5* knock-out did not abolish the interaction between pre-IL-1α and either histone acetyltransferase complex because the interaction complexes were visible in the co-immunoprecipitation experiments in the *gcn5*Δ yeast strains producing either the Ahc1-TAP or Spt8-TAP proteins. This result strongly suggests that the IL-1α precursor does not interact with the catalytic subunit of the HAT/Core module, and the IL-1α-binding site should thus comprise Ada2, Ada3 and Sgf29 only (Figure 6A). As expected, the disruption of the *SPT7* gene, which encodes a subunit that is important for the maintenance of the integrity of the SAGA complex, did not affect the interaction between the IL-1α precursor and the ADA complex, as represented by the Ahc1 protein, but the disruption completely abolished the co-immunoprecipitation of pre-IL-1α with the Spt8 protein (Figure 6C).

In agreement with our results and expectations, the deletion of the *AHC1* gene, which is a marker of the ADA complex, did not disrupt the co-immunoprecipitation of pre-IL-1α with the Gcn5 catalytic subunit and the Spt8 protein, thus suggesting that the IL-1α precursor remained associated with the SAGA complex. Surprisingly, we were only rarely able to detect the Spt7-TAP protein in a complex with pre-IL-1α from the TAP/Spt7,*ahc1*Δ yeast strain. If obtained, the signal was weak and the success rate of detectable co-immunoprecipitation of Spt7-TAP with pre-IL-1α in the *ahc1*Δ strain was 1 in 4 cases (Figure 6B and S2). Because Ahc1 and Spt7 proteins have not been found together in any HAT complex, this result, together with the genetic and biochemical studies performed by our group and others, suggests a novel model of ADA function in SAGA complex assembly in which the binding of Spt7 and Ahc1 are mutually exclusive. This hypothesis is further supported by confirmation that the disruption of the *AHC1* gene does not significantly affect the intracellular level of the Spt7 protein (Figure S2). The inability to co-immunoprecipitate Spt7-TAP with pre-IL-1α cannot also be a result of an inefficient western blotting, because in case of TAP/Spt7,*ahc2*Δ strain (Figure 6A) and TAP/Spt7 strain alone (Figure 5), the Spt7 protein was detected in all cases even from less yeast cells. Further, as we show in Figure S2, Spt7 proteins were detected readily in all input lines which were resolved in the same gel as IP samples from TAP/Spt7,*ahc1*Δ strain.

## Discussion

The heterologous expression of various proteins in *Saccharomyces cerevisiae* as a model organism has been widely used for the assessment of protein function and characteristics for many years. A large selection of yeast strains and various promoters and the relative ease of genetic manipulation make this model a useful and versatile tool in biology. We have been using the yeast *Saccharomyces cerevisiae* as a model organism to study the nuclear function of the human proinflammatory cytokine IL-1α. Previously, we had shown an unexpected genetic interaction between IL-1αNTP and the yeast histone acetyltransferase complexes by demonstrating that the Gal4BD/IL-1αNTP fusion protein transactivates transcription in the UAS/GAL system in cells containing an intact SAGA HAT complex. The overexpression of the Gal4BD/IL-1αNTP fusion induced growth inhibition, which was attenuated in strains bearing a deletion of an adaptor (Ada2, Ada3), enzymatic (Gcn5) or structural (Spt7) SAGA subunit, whereas the deletion of Ahc1 (the critical structural subunit of the ADA complex) had no effect, thus resembling a classical Ada^-^ phenotype. Importantly, we were able to confirm all of the functional and genetic interactions of IL-1αNTP, including studies with its various deletion mutants, with yeast SAGA complex in the mammalian system and provided evidence that IL-1αNTP physically and functionally interacts with human PCAF/p300/Gcn5 histone acetyltransferase complexes, thus obtained experimental proof that the putative IL-1αNTP-binding site in the SAGA complex is evolutionarily conserved from yeast to humans [40]. These findings and knowledge that the SAGA complex is well conserved across all eukaryotes and currently serves as an archetype of the histone acetyltransferase complexes encouraged us to utilize the yeast SAGA complex as a model system for investigating IL-1αNTP interaction with the HAT complexes. Our study was further stimulated with knowledge that other mammalian transcription factors were shown to behave similarly and interact with homologous proteins and structures in yeast and humans. A privilege position among them holds the p53 protein. Analysis of the influence of chromatin-remodeling and histone-modifying complexes on p53-dependent activation of transcription in yeast revealed many similarities between transcriptional control by p53 in yeast and human cells [53], and nowadays yeast serves as a valuable tool for functional analysis of separated tumor-derived p53 alleles (FASAY) [54], [55], [56].

Despite an increasing number of studies dedicated to the nuclear function of IL-1αNTP and the IL-1α precursor, the structure of these molecules is yet to be determined. Therefore, we predicted the IL-1αNTP structure using the Robetta server and confirmed the resulting structures by performing similarity searches with the known protein structures in the Protein Data Bank. We hypothesized that if a putative IL-1αNTP-binding pocket in the SAGA complex is evolutionarily conserved and if the binding of IL-1αNTP to the corresponding human HAT complex has biological function, a yeast protein may exist that contains a domain that is structurally homologous to human IL-1αNTP. We found such a domain within the C-terminal region of the Snf1 protein kinase. The Snf1 kinase is a highly evolutionarily conserved AMP-activated serine/threonine protein kinase that serves as a cellular energy sensor. Interestingly, the Snf1 protein kinase was shown to interact with several proteins constituting both the SAGA and ADA complexes. Among these proteins, Snf1 phosphorylates Gcn5 and is regulated by Ubp8-mediated deubiquitination [47], [48], [49], [50]. We found that 3-AT hypersensitivity of the *snf1*Δ yeast strains can be partially rescued by overexpression of human pre-IL-1α, which provides an experimental evidence for the biological relevance of predicted structural similarity between the IL-1αNTP and Snf1 C-terminal domain. Interestingly the highest suppression of the 3-AT toxicity we observed in the yeast strain lacking *SNF1* gene but the N-terminal 108 codons [48] growing on agar media containing 10 mM 3-AT and glycerol as a carbon source. This has a sense, because Snf1 kinase is, besides other stimuli, activated during amino acid and generally nitrogen starvation [57], [58] and during the shift from glucose to glycerol media, when its relocation to the nucleus was also reported [59]. Further, the N-terminal 108 amino acids contain almost complete a structurally isolated β-rich lobe of the Snf1 kinase domain comprising of β1-3 strands and a well conserved regulatory αC helix including ATP-binding site and conserved residues Lys84 and Glu103. This part of Snf1 is structurally similar to the cyclin-binding domain of cyclin-dependent kinase 2 (CDK2) [60] which points to the possibility that even such a small portion of the Snf1 protein may have a potential to interact with other proteins and show some biological function if artificially expressed in the yeast cell alone. By chance, this happened on glycerol media when we screened for conditions, in which pre-IL-1α would show the highest suppression of *snf1*Δ strain hypersensitivity to 3-AT inhibitor. This result further points to the evolutionary conservation of the Snf1/AMPK signaling pathway, which has been evidenced in the past e.g. by discovery of mammalian TAK1 (transforming growth factor-β-activated kinase) as the AMPK-activating kinase using mutant yeast strains lacking all three Snf1-activating kinases [61], and thus also further rationalizes the usage of the yeast model for investigation of the nuclear function of pre-IL-1α.

The C-terminal regulatory region of the catalytic subunit of the mammalian AMP-activated protein kinase contains a domain that is structurally almost identical to that of Snf1, which we termed the Interleukin-1αNTP-Like domain (INL domain). Mammalian AMPK is at the center of the regulation of cellular energy homeostasis. However, AMPK is also activated by a number of extracellular signals, including cytokines, drugs and polyphenols. The downstream processes regulated by mammalian AMPK include glucose transport, transcription, cell growth and proliferation and cell polarity maintenance. Several transcription factors and co-activators, including p300, serve as direct AMPK targets [50], [62]. Previously, we performed a larger deletion study of p300 in human embryonic kidney 293 cells (HEK293) to determine the region responsible for p300- and Gal4BD/IL-1αNTP-dependent transactivation of the Gal4 reporter system. Interestingly, one of the two most active p300 mutants (Δ143-957) lacked a large portion of the N-terminal region, but the N-terminal 142 amino acid residues containing also an AMPK phosphorylation site at Ser89 [40]. Taking all of these data together, it is tempting to speculate that mammalian AMPK can compete with IL-1αNTP or the IL-1α precursor for the same binding pocket in the histone acetyltransferase complexes. We also obtained solid initial data suggesting that the C-terminal INL domain of yeast and mammalian Snf1/AMPK kinases may directly interact with their own N-terminal β-rich lobe where it may compete with other INL domain-containing proteins, the mammalian interleukin-1α precursor included.

In a substantial portion of the study, we focused on identifying of an IL-1α precursor-binding site in the SAGA complex. We employed a library of yeast strains that stably express genes coding for TAP-tagged SAGA and ADA subunits in place of their endogenous counterparts. We further extended this library by performing deletions of genes encoding the catalytic and structural HAT complex subunits (*gcn5*Δ, *ahc1*Δ, *ahc2*Δ and *spt7*Δ) in selected TAP-tagged yeast strains. The immunoprecipitation of the IL-1α precursor followed with western blotting with an anti-CBP antibody that identifies the TAP-tagged proteins allowed us to dissect the HAT subcomplexes interacting with the IL-1α precursor in more detail. We found that the IL-1α precursor co-precipitates not only with the expected subunits of the SAGA complex but also with Ahc1, which is a marker of the ADA complex. This result was surprising because our previous work did not show any changes in the Gal4BD/IL-1αNTP transactivation potential in *ahc1* null mutant strains, which was in contrast to results from *ada2, ada3, gcn5* and *spt7* knock-outs [40]. This finding restricted the IL-1αNTP-binding site to the HAT/Core module because this is the only module shared by both the ADA and SAGA complexes [7], [10]. The co-immunoprecipitation experiments in the TAP/Ahc1,*gcn5*Δ and TAP/Spt8,*gcn5*Δ strains further excluded Gcn5 as a candidate for IL-1α binding. These results suggest that the IL-1αNTP/pre-IL-1α-binding site in the SAGA/ADA complexes is formed by Ada2, Ada3, Sgf29 and perhaps other non-canonical HAT/Core proteins. We also cannot exclude that another yeast HAT complex called SLIK/SALSA (SAGA-like/SAGA altered, Spt8 absent) may bind pre-IL-1α because it shares the same HAT/Core subunits with SAGA and ADA, and the only difference in this complex is the presence of a truncated form of Spt7 and the lack of Spt8 [63]. The presence of Spt8 in the samples after immunoprecipitation could thus mask the SLIK/SALSA complex.

**Figure 7:**
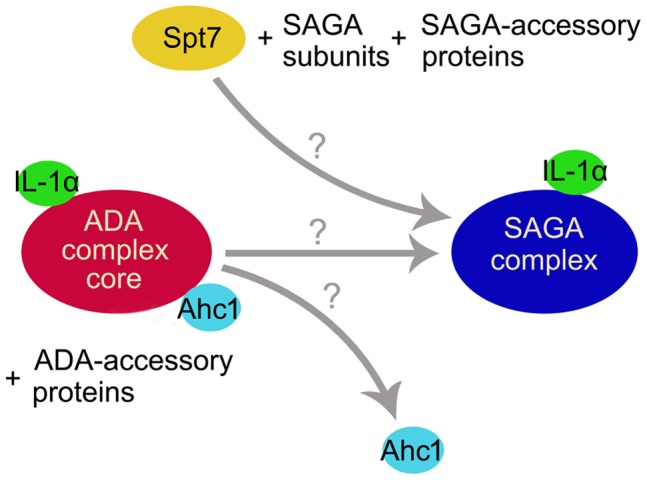
A model suggesting a mutually exclusive role for Ahc1 and Spt7 in SAGA complex assembly. Co-IP experiments showed that pre-IL-1α binds to the HAT/Core of both the ADA and SAGA complexes. In the TAP/Spt7,*ahc1*Δ strain, only rarely weak co-precipitation of Spt7-TAP and pre-IL-1α was observed. Ahc1 thus may operate as an exchange factor that facilitates Spt7 binding to the ADA HAT, bringing various non-canonical co-activators and accessory proteins (e.g., IL-1α) and providing the resulting complex with DNA-binding abilities that give rise to a fully functional SAGA complex. Therefore, at least from the point of IL-1α function, ADA might not represent a real HAT complex but rather an intermediate protein complex that is however necessary for the assembly and proper function of the SAGA HAT complex.

Analysis of selected TAP-tagged yeast strains carrying deletion of the *SPT7* and *AHC1* genes, which are believed to maintain the integrity of the SAGA and ADA complex, respectively [7], [26], revealed that although the loss of Spt7 from the cell leads to the disintegration of the SAGA but not the ADA complex, as expected, the loss of Ahc1 from the cell did not allow for the efficient co-immunoprecipitation of the IL-1α precursor with Spt7-TAP. However, an *ahc1* null mutation did not affect the co-immunoprecipitation of the IL-1α precursor and Gcn5, a constituent of the HAT/Core, and Spt8, an exclusive member of the SAGA complex (Figure 6). To exclude that the results are influenced by disturbances either in the Spt7 protein levels in the *ahc1*Δ strain or in the experimental procedure, we repeated the experiment several times and confirmed by western blotting that *ahc1* deletion does not significantly reduce intracellular Spt7 protein levels and also immunoprecipitation of the IL-1α precursor from the corresponding yeast cell lysates worked well (Figure S2). These results are unexpected for several reasons. First, Spt7 is the core SAGA subunit that is present even in the absence of Spt3, Spt8, Spt20, Gcn5 and Ada1 [64]. In the absence of Spt7, the SAGA HAT complex is disrupted, and this disruption severely affects transcriptional activation [7], [65]. Spt7 also regulates the levels of certain SAGA subunits and plays a central role in the SAGA complex formation [64]. Thus, the reason why Spt7 should be absent in a protein complex co-immunoprecipitated with pre-IL-1α in the *ahc1* deletion strain is not obvious unless the ADA complex is the complex responsible. Furthermore, Spt8 is believed to be closely associated with Spt7, and its interaction with SAGA has been reported to be dependent on Spt7 [10], [64]. These results may be explained by a new model of Ahc1 function in yeast cells in which Ahc1 contributes to the association of Spt7 with SAGA. We speculate that Ahc1 functions as an exchange factor that is not exclusively required for but facilitates the association of Spt7 and perhaps other factors with the ADA HAT complex, resulting in a fully functional SAGA complex that is capable of interacting with various general and non-canonical transcriptional co-activators and accessory proteins, such as pre-IL-1α and AMP-activated protein kinase. Therefore, ADA would, at least for some of the cellular regulatory loops, not represent a HAT complex but rather an intermediate and/or reserve protein complex that is associated with non-canonical co-activators and other accessory proteins that may be necessary for the proper assembly of the SAGA complex with all of its co-activators. It should be noted that the purification of the SAGA complex has also been reported from the *ahc1*Δ strain [26]. However, the proposed model does not exclude ADA-independent SAGA assembly but rather suggests that some co-activators or accessory proteins may be brought to the SAGA complex only via the ADA complex, which may also have a regulatory function in the control of gene expression (Figure 7). This model is supported by recent data presented by Lee and co-workers, who dissected SAGA and ADA complexes using systematic gene knock-out and TAP-mediated protein complex purification approaches. They demonstrated that using Ada2-TAP, they could, as one would expect, purify most of the SAGA or ADA complex subunits in different knock-out strains with the exception of TAP-Ada2/*spt20*Δ and TAP-Ada2/*ahc2*Δ. In the first case, only the ADA complex was present, and in the latter case, only the HAT/Core module could be precipitated using a TAP tag. Lee and co-workers suggested that Ahc2 is responsible for tethering Ahc1 into the Ada complex [10]. Their TAP-Ada2/*ahc2*Δ result would be in agreement with the results of our experiments in which Spt7 did not efficiently co-purify with pre-IL-1α in the *ahc1*Δ strain. However, in our hands, pre-IL-1α readily co-immunoprecipitated with Gcn5-TAP, Spt8-TAP and Spt7-TAP in the corresponding *ahc2*Δ strains. There can be more explanations of this result, including that we tested different TAP-tagged proteins, used yeast with a different genetic background and namely immunoprecipitated the HAT complexes via pre-IL-1α, which may also have some, yet unknown function in the HAT complex assembly. Should be stressed, that just few our and Washburn’s group experiments, together concerning the Ahc2 function, provided the first results about this only recently annotated protein and more work focused on elucidation of Ahc2 role in the ADA complex assembly and activity will have to be done. To the best of our knowledge, the enzymatic activities and substrate specificity of the ADA complex have only been determined in vitro. Moreover, both Lee and co-workers [10] and Eberharter and co-workers [26] obtained different results, which may be explained by different methods of ADA complex purification. On the other hand, we and both of these groups demonstrated that the knock-out of the *AHC1* gene, which is specific for the ADA complex, cannot rescue the Gal4BD-VP16- or Gal4BD-IL-1αNTP-mediated toxicity [10], [26], [40], suggesting a low direct transactivation potential of the ADA complex.

Last but not least, it should be noted that the subunit composition of yeast histone acetyltransferase complexes may vary, which is the case for SAGA and another yeast HAT complex SLIK/SALSA [63], [65]. Variable subunits, co-activators and other accessory proteins may participate in the assembly of yeast histone acetyltransferase complexes, making it difficult to precisely define their subunit composition. Likewise, the precise physiological roles of the various histone acetyltransferase complexes remain to be uncovered in yeast and humans.

## Supporting Information

Figure S1 Expression and immunoprecipitation of IL-1αMat from the TAP-fusion strains. (A) Expression of the TAP-fused HAT complex subunits in the corresponding yeast cell lysates used as an input for the immunoprecipitation of mature IL-1α. The expression of all of the subunits tested was confirmed with western blotting with an anti-CBP antibody that recognizes the TAP tag at the C-terminus of the respective HAT complex subunits. (B) Mature IL-1α immunoprecipitates from the lysates of the S. cerevisiae strains expressing TAP-tagged HAT complex subunits. Western blotting was performed using an anti-Flag antibody that recognizes the Flag tag at the N-terminus of mature IL-1α (Mat). The asterisk indicates the band corresponding to the light chain of the anti-Flag antibody. Molecular size marker positions are shown at the right.
(TIF)

Figure S2 Co-immunoprecipitation of Spt7-TAP with pre-IL-1α from the TAP/Spt7 and TAP/Spt7,ahc1Δ strains. (A) Strain TAP/Spt7; Spt7-TAP co-precipitated with both Flag-pre-IL-1α and Flag-pre-IL-1α-HA produced independently in this strain. Primary antibody: anti-CBP, secondary antibody: swine anti-rabbit. (B) Strain TAP/Spt7,ahc1Δ; no Spt7-TAP could be co-precipitated neither with Flag-pre-IL-1α nor with Flag-pre-IL-1α-HA from the TAP/Spt7,ahc1Δ strain in this experiment. After several rounds of experiments we were able to obtain a weak signal of Spt7-TAP in lysates from TAP/Spt7,ahc1Δ in one of four experiments in average (see Figure 6). As it is clearly seen from input lines of all experiments, the disruption of the AHC1 gene does not significantly affect the intracellular levels of the Spt7 protein. Primary antibody: anti-CBP, secondary antibody: swine anti-rabbit. Staining with anti-Flag antibody in the last panel confirmed successful pre-IL-1α immunoprecipitation from the TAP/Spt7,ahc1Δ lysates used in the experiment; primary antibody: mouse anti-Flag, secondary antibody: goat anti-mouse.
(TIF)

File S1 Prediction of the 3-D structure of the first N-terminal 112 amino acid residues of the IL-1α precursor (IL-1αNTP). (Coordinates in PDB format)
(PDB)

File S2 SNF1, AMPK and IL-1α sequences used for protein structures modeling and comparison.
(PDF)
