# Supplementary figures and images for: N-Terminal Domain of Nuclear IL-1α Shows Structural Similarity to the C-Terminal Domain of Snf1 and Binds to the HAT/Core Module of the SAGA Complex
Source: PLoS One. 2012 Aug 6;7(8):e41801. doi: 10.1371/journal.pone.0041801 (PMC3412866; doi:10.1371/journal.pone.0041801)

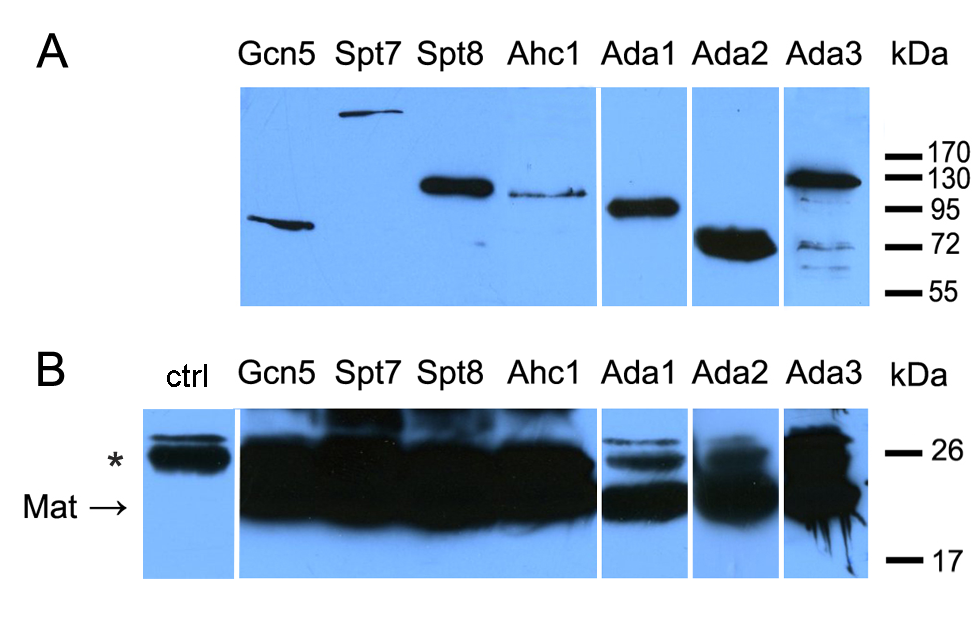

Supplement: Figure S1 — Expression and immunoprecipitation of IL-1αMat from the TAP-fusion strains. (A) Expression of the TAP-fused HAT complex subunits in the corresponding yeast cell lysates used as an input for the immunoprecipitation of mature IL-1α. The expression of all of the subunits tested was confirmed with western blotting with an anti-CBP antibody that recognizes the TAP tag at the C-terminus of the respective HAT complex subunits. (B) Mature IL-1α immunoprecipitates from the lysates of the S. cerevisiae strains expressing TAP-tagged HAT complex subunits. Western blotting was performed using an anti-Flag antibody that recognizes the Flag tag at the N-terminus of mature IL-1α (Mat). The asterisk indicates the band corresponding to the light chain of the anti-Flag antibody. Molecular size marker positions are shown at the right. (TIF) [file pone.0041801.s001.tif]

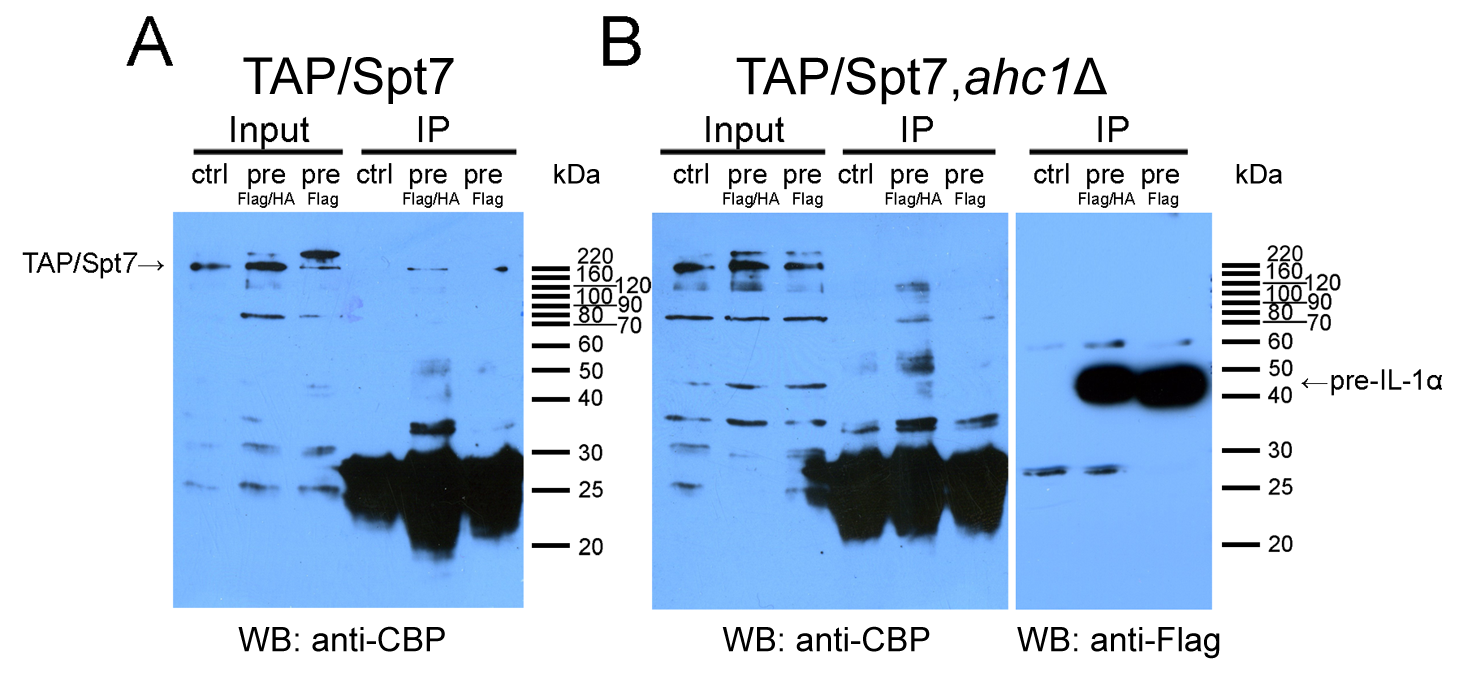

Supplement: Figure S2 — Co-immunoprecipitation of Spt7-TAP with pre-IL-1α from the TAP/Spt7 and TAP/Spt7,ahc1Δ strains. (A) Strain TAP/Spt7; Spt7-TAP co-precipitated with both Flag-pre-IL-1α and Flag-pre-IL-1α-HA produced independently in this strain. Primary antibody: anti-CBP, secondary antibody: swine anti-rabbit. (B) Strain TAP/Spt7,ahc1Δ; no Spt7-TAP could be co-precipitated neither with Flag-pre-IL-1α nor with Flag-pre-IL-1α-HA from the TAP/Spt7,ahc1Δ strain in this experiment. After several rounds of experiments we were able to obtain a weak signal of Spt7-TAP in lysates from TAP/Spt7,ahc1Δ in one of four experiments in average (see Figure 6). As it is clearly seen from input lines of all experiments, the disruption of the AHC1 gene does not significantly affect the intracellular levels of the Spt7 protein. Primary antibody: anti-CBP, secondary antibody: swine anti-rabbit. Staining with anti-Flag antibody in the last panel confirmed successful pre-IL-1α immunoprecipitation from the TAP/Spt7,ahc1Δ lysates used in the experiment; primary antibody: mouse anti-Flag, secondary antibody: goat anti-mouse. (TIF) [file pone.0041801.s002.tif]
