# Supplementary material for: N-Terminal Domain of Nuclear IL-1α Shows Structural Similarity to the C-Terminal Domain of Snf1 and Binds to the HAT/Core Module of the SAGA Complex
Source: PLoS One. 2012 Aug 6;7(8):e41801. doi: 10.1371/journal.pone.0041801 (PMC3412866; doi:10.1371/journal.pone.0041801)
Supplement: File S2 — SNF1, AMPK and IL-1α sequences used for protein structures modeling and comparison. (PDF) [file pone.0041801.s004.pdf]

SNF1, 3T4N, (AAB64904.1), aa 505--630 were used for the model

>gi|927732|gb|AAB64904.1| Snf1p: serine/threonine protein kinase [Saccharomyces cerevisiae]
mssnnntntapananssshhhhhhhhhhhhhghggsnstlnnpkssladgahignyqivktlgegsfgkvklayhtttgqk
valkiinkkvlaksdmqgriereisylrllrhphiiklydvikskdeiimvieyagnelfdyivqrdkmseqearrffqq
iisaveychrhkivhrdlkpenllldehlnvkiadfglsnimtdgnflktscgspnyaapevisgklyagpevdvwscgv
ilyvmlcrrlpfddesipvlfknisngvytlpkflspgaaglikrmlivnplnrisiheimqddwfkvdlpeyllppdlk
phpeeenenndskkdgsspdndeiddnlvnilsstmgyekdeiyeslessedtpafneirdaymlikenkslikdmkank
svsdeldtflsqspptfqqqskshqksqvdhetakqharrmasaitqqrtyhqspfMDQYKEEDSTVSILPTSLPQIHRA
NMLAQGSPAASKISPLVTKKSKTRWHFGIRSRSYPLDVMGEIYIALKNLGAEWAKPSEEDLWTIKLRWKYDIGNKTNTNE
KIPDLMKMVIQLFQIETNNYLVDFKFDGWESSYGDDTTVSNISEDEMSTFSAYPFLHLTTKLIMELAVNSQSN

>3T4N:A|PDBID|CHAIN|SEQUENCE (used for crystallization and modeling)
gpMDQYKEEDSTVSILPTSLPQIHRANMLAQGSPAASKISPLVTKKSKTRWHFGIRSRSYPLDVMGEIYIALKNLGAEWA
KPSEEDLWTIKLRWKYDIGNKTNTNEKIPDLMKMVIQLFQIETNNYLVDFKFDGWESSYGDDTTVSNISEDEMSTFSAYP
FLHLTTKLIMELAVNSQSN

AMPK, 2V92 (P54645.2), aa 406--559 were used for the model

>gi|254763245|sp|P54645.2|AAPK1_RAT RecName: Full=5'-AMP-activated protein kinase catalytic subunit alpha-1; Short=AMPK subunit alpha-1; AltName: Full=Tau-protein kinase PRKAA1
mrrlsswrkmataekqkhdgrvkighyilgdtlgvgtfgkvkvgkheltghkvavkilnrqkirsldvvgkirreiqnlk
lfrhphiiklyqvistpsdifmvmeyvsggelfdyickngrldekesrrlfqqilsgvdychrhmvvhrdlkpenvllda
hmnakiadfglsnmmsdgeflrtscgspnyaapevisgrlyagpevdiwssgvilyallcgtlpfdddhvptlfkkicdg
ifytpqylnpsvisllkhmlqvdpmkratikdirehewfkqdlpkylfpedpsysstmiddealkevcekfecseeevls
clynrnhqdplavayhliidnrrimneakdfylatsppdsflddhhltrphpervpflvaetprarhtldelnpqkskhq
gvrkakwHLGIRSQSRPNDIMAEVCRAIKQLDYEWKVVNPYYLRVRRKNPVTSTFSKMSLQLYQVDSRTYLLDFRSIDDE
ITEAKSGTATPQRSGSISNYRSCQRSDSDAEAQGKPSEVSLTSSVTSLDSSPVDVAPRPGSHTIEFFEMCANLIKILAQ

>2V92:A|PDBID|CHAIN|SEQUENCE (used for crystallization and modeling)
gsmawHLGIRSQSRPNDIMAEVCRAIKQLDYEWKVVNPYYLRVRRKNPVTSTFSKMSLQLYQVDSRTYLLDFRSIDDEIT
EAKSGTATPQRSGSISNYRSCQRSDSDAEAQGKPSEVSLTSSVTSLDSSPVDVAPRPGSHTIEFFEMCANLIKILAQ

IL-1alpha N-terminal domain (prediction)

>gi|27894330|ref|NP_000566.3| interleukin-1 alpha proprotein [Homo sapiens]
MAKVPDMFEDLKNCYSENEEDSSSIDHLSLNQKSFYHVSYGPLHEGCMDQSVSLSISETSKTSKLTFKESMVVVATNGKV
LKKRRLSLSQSITDDDLEAIANDSEEEIIKPRsapfsflsnvkynfmriikyefilndalnqsiirandqyltaaalhnl
deavkfdmgayksskddakitvilrisktqlyvtaqdedqpvllkempeipktitgsetnllffwethgtknyftsvahp
nlfiatkqdywvclaggppsitdfqilenqa

Legend:

Lower-case – aa not used for crystallization or model prediction or does not correspond to original protein sequence

correct position of the residue was not determined and/or depicted

Alpha-helix

Beta-sheet

References:

Xiao B, Heath R, Saiu P, Leiper FC, Leone P, Jing C, Walker PA, Haire L, Eccleston JF, Davis CT, Martin SR, Carling D, Gamblin SJ., (2007) Structural basis for AMP binding to mammalian AMP-activated protein kinase. Nature. 2007 Sep 27;449(7161):496-500. Epub 2007 Sep 12.

Mayer FV, Heath R, Underwood E, Sanders MJ, Carmena D, McCartney RR, Leiper FC, Xiao B, Jing C, Walker PA, Haire LF, Ogrodowicz R, Martin SR, Schmidt MC, Gamblin SJ, Carling D.(2011) ADP regulates SNF1, the Saccharomyces cerevisiae homolog of AMP-activated protein kinase. Cell Metab. 2011 Nov 2;14(5):707-14. Epub 2011 Oct 20.
